# Combined Red Cell Exchange and Plasma Exchange for Post-Arrest Refractory Cardiorespiratory Failure in Sickle Cell Disease Sparing Extracorporeal Membrane Oxygenation

**DOI:** 10.14740/jmc5350

**Published:** 2026-07-28

**Authors:** Ashraf Ezzat Attia

**Affiliations:** Department of Critical Care, College of Medicine Imam Abdulrahman Bin Faisal University Dammam, Saudi Arabia. Email: aattia@iau.edu.sa

**Keywords:** Acute chest syndrome, Exchange transfusion, Plasma exchange, Sickle cell disease, Extracorporeal membrane oxygenation, Therapeutic apheresis, Multiorgan failure, fat embolism syndrome

## Abstract

Extracorporeal membrane oxygenation (ECMO) is increasingly utilized for refractory respiratory failure in sickle cell disease (SCD), yet registry data indicate in-hospital survival of only 40% in adults, with particular concerns regarding hemolysis, thrombosis, and bleeding processes already pathologically amplified in SCD. A targeted hematologic strategy using combined therapeutic plasma exchange (PLEX) and red cell exchange (RCE) may offer an alternative approach, but high-quality outcome data remain limited. We report a 37-year-old male with homozygous SCD (HbSS genotype) and glucose-6-phosphate dehydrogenase (G6PD) deficiency who presented with severe vaso-occlusive crisis that progressed to two cardiac arrests, shock requiring four vasopressors, severe acute respiratory distress syndrome (PaO_2_/FiO_2_ 56 mm Hg), biventricular failure, and multiorgan dysfunction. Laboratory findings included marked lactate dehydrogenase elevation (7,169 U/L, > 25 × upper limit of normal) and severe thrombocytopenia (platelet count 37 × 10^3^/µL). A multidisciplinary team considered ECMO but elected to pursue combined RCE and daily PLEX as salvage therapy. Seven sessions of daily PLEX combined with intermittent RCE reduced the hemoglobin S fraction from 73% to 18%. Vasopressor requirements decreased within 24 h, with complete independence by day 8. Inflammatory markers declined substantially within 72 h. The patient was extubated on day 10 and discharged from the intensive care unit on day 14 without neurological deficit. This case demonstrates that combined RCE and daily PLEX may represent a viable salvage strategy for post-arrest refractory cardiorespiratory failure in SCD, potentially sparing the need for ECMO in carefully selected patients. Prospective validation is essential.

## Introduction

Sickle cell disease (SCD) affects approximately 100,000 individuals in the United States and 300,000 births annually worldwide, representing one of the most common monogenic disorders globally [1, 2]. Despite therapeutic advances, acute complications remain life-threatening, with acute chest syndrome (ACS) representing the leading cause of mortality in adult patients [3, 4].

ACS is a clinicoradiological syndrome characterized by new pulmonary infiltrates, respiratory symptoms, fever, and hypoxemia occurring in the context of SCD [5]. The pathophysiology is multifactorial, encompassing infection, pulmonary infarction, fat embolism from bone marrow necrosis, and inflammatory lung injury [6, 7]. Progression to acute respiratory distress syndrome (ARDS) and multi-organ dysfunction occurs in 10–20% of severe cases, with mortality exceeding 20% despite optimal care [8].

The management of refractory respiratory failure in SCD has increasingly incorporated extracorporeal membrane oxygenation (ECMO); however, systematic analyses reveal inconsistent outcomes, with particular concerns regarding hemolysis, thrombosis, bleeding complications, and inflammatory activation—processes already pathologically amplified in SCD [9, 10]. Registry analyses of 206 adult SCD patients receiving ECMO report overall in-hospital survival of only 40.3%, with venoarterial ECMO survival at 25.5% versus venovenous ECMO at 61.1% [9]. Hemorrhage occurred in 26% of patients, neurological injury in 22%, and thrombosis in 13% [9, 10].

Therapeutic plasma exchange (PLEX) has emerged as a potential adjunct in severe SCD complications based on its ability to remove circulating cytokines, free hemoglobin, immune complexes, and ultra-large von Willebrand factor multimers implicated in microvascular thrombosis [11, 12]. The American Society for Apheresis (ASFA) provides a grade 2B recommendation for PLEX in SCD with multiorgan failure, though high-quality outcome data remain limited [11].

We report a case of fulminant SCD crisis with refractory cardiorespiratory failure in which a combined red cell exchange (RCE)-PLEX strategy achieved rapid reversal of multi-organ dysfunction, sparing the need for ECMO despite initial multidisciplinary consensus favoring mechanical support.

## Case Report

### Patient information

A 37-year-old male with homozygous SCD (HbSS genotype) and glucose-6-phosphate dehydrogenase (G6PD) deficiency presented with severe vaso-occlusive crisis involving the chest, back, and lower extremities. His disease history was marked by high acuity, with seven annual hospital admissions, two prior intensive care unit admissions for ACS, chronic avascular necrosis of the femoral heads (status post bilateral total hip arthroplasty), chronic recurrent multifocal osteomyelitis, multiple vertebral compression fractures, iron overload (ferritin > 2,500 ng/mL) from recurrent transfusions, cholelithiasis (status post cholecystectomy), and recent hemorrhoidectomy (6 weeks prior). Baseline functional status was Modified Rankin Scale 2 (independent but limited by chronic pain). Relevant family history included sickle cell trait in both parents. The patient was a non-smoker and worked in administrative support. His primary concern at presentation was uncontrolled pain and progressive difficulty breathing. The patient had no known baseline cardiac disease, including congestive heart failure, pulmonary hypertension, or arrhythmia.

### Timeline

On admission (day 0, September 21, 2025) (Table 1), the patient was alert and oriented with stable vital signs. Pulse oximetry demonstrated SpO_2_ 94% on room air. A portable chest radiograph showed bilateral lower lobe infiltrates consistent with evolving ACS. Empiric broad-spectrum antibiotics were not initiated at this time, as the patient was hemodynamically stable without clear evidence of bacterial infection, and the elevated procalcitonin (2.1 ng/mL; reference < 0.05 ng/mL) and white blood cell count (12.3 × 10^3^/µL; reference 4.5–11.0 × 10^3^/µL) were attributed to ACS and vaso-occlusive crisis. Significant findings included pallor and diffuse tenderness over the chest, spine, and bilateral lower extremities without joint swelling. Lung auscultation revealed decreased breath sounds at bilateral bases. He was admitted to the ward for standard supportive care including intravenous hydration, opioid analgesia, and incentive spirometry.

**Table 1:** Timeline of Clinical Events

Date/day	Event
Day 0	Admission to ward for vaso-occlusive crisis; supportive care initiated
Day 1 (morning)	Progressive dyspnea and hypoxemia; BiPAP initiated
Day 1 (afternoon)	ICU transfer; hypotension requiring norepinephrine; PEA arrest with ROSC after 6 min CPR
Day 1 (evening)	SVT to AF with RVR; two synchronized cardioversions; recurrent VT; three defibrillations + 6 min CPR; second ROSC
Day 1 (post-arrest)	Shock with four vasopressors; severe ARDS (PaO_2_/FiO_2_ 56); biventricular failure on echo; GCS 12; multidisciplinary decision to initiate combined RCE and PLEX; ECMO circuit prepared but not initiated
Day 2	First PLEX session; first RCE session; vasopressor requirements begin to decrease
Days 2–8	Daily PLEX (seven total sessions); intermittent RCE every 48 h
Day 3	HbS 35%; inflammatory markers declining
Day 8	Complete vasopressor independence
Day 10	Repeat echocardiography showing normalized LVEF and improved RV function; platelet count normalized; extubation to pressure support ventilation (PaO_2_/FiO_2_ 491)
Day 14	ICU discharge; full neurologic recovery
Day 16	Hospital discharge to home

AF: atrial fibrillation; ARDS: acute respiratory distress syndrome; BiPAP: bilevel positive airway pressure; CPR: cardiopulmonary resuscitation; FiO_2_: fraction of inspired oxygen; GCS: Glasgow Coma Scale; HbS: hemoglobin S (sickle hemoglobin); ICU: intensive care unit; LVEF: left ventricular ejection fraction; PaO_2_: partial pressure of oxygen in arterial blood; PEA: pulseless electrical activity; PLEX: plasma exchange; RCE: red cell exchange; ROSC: return of spontaneous circulation; RVR: rapid ventricular response; RV: right ventricular; SVT: supraventricular tachycardia; VT: ventricular tachycardia.

On day 1 (September 22, 2025), he deteriorated rapidly with progressive dyspnea and hypoxemia (SpO_2_ 88% on room air). Within 4 h of intensive care unit admission, the patient became hypotensive requiring norepinephrine infusion, followed by progressive bradycardia leading to pulseless electrical activity arrest. Return of spontaneous circulation was achieved after 6 min of cardiopulmonary resuscitation per ACLS protocol. Post-arrest, he developed sustained supraventricular tachycardia degenerating to atrial fibrillation with rapid ventricular response, requiring two synchronized cardioversions (100 J, 150 J). Several hours later, recurrent hemodynamic collapse with unstable ventricular tachycardia necessitated three defibrillations and 6 min of cardiopulmonary resuscitation.

Following the second arrest (September 23, 2025), the patient was in shock requiring four vasopressors (norepinephrine, epinephrine, vasopressin, dobutamine). Physical examination revealed cool extremities, mottled skin, and Glasgow Coma Scale 12 (E3, V4, M5). Cardiac auscultation demonstrated tachycardia with an irregular rhythm. Pulmonary examination revealed diffuse crackles bilaterally with poor air entry at the bases. Abdominal examination was benign.

Admission laboratory values were as follows: hemoglobin 7.8 g/dL (reference 13.5–17.5 g/dL), hemoglobin S 73%, platelets 145 × 10^3^/µL (reference 150–400 × 10^3^/µL), white blood cells 12.3 × 10^3^/µL (reference 4.5–11.0 × 10^3^/µL), lactate dehydrogenase (LDH) 2,150 U/L (reference 140–280 U/L), total bilirubin 4.2 mg/dL (reference 0.1–1.2 mg/dL), direct bilirubin 0.9 mg/dL (reference 0.0–0.3 mg/dL), troponin I 0.04 ng/mL (reference < 0.04 ng/mL), procalcitonin 2.1 ng/mL (reference < 0.05 ng/mL), C-reactive protein (CRP) 16 mg/L (reference < 5 mg/L), international normalized ratio (INR) 1.2 (reference 0.9–1.1), prothrombin time (PT) 12 s (reference 11–13.5 s), activated partial thromboplastin time (aPTT) 32 s (reference 25–35 s), fibrinogen 3.2 g/L (reference 2.0–4.0 g/L), lactate 1.2 mmol/L (reference 0.5–2.2 mmol/L), creatinine 0.67 mg/dL (reference 0.7–1.3 mg/dL), blood urea nitrogen (BUN) 28 mg/dL (reference 7–20 mg/dL), and pH 7.38 (reference 7.35–7.45). Arterial blood gas on admission was not obtained as the patient was not hypoxemic.

On day 1, values worsened: hemoglobin 7.7 g/dL (reference 13.5–17.5 g/dL), platelets 84 × 10^3^/µL (reference 150–400 × 10^3^/µL), white blood cells 12.7 × 10^3^/µL (reference 4.5–11.0 × 10^3^/µL), LDH 3,855 U/L (reference 140–280 U/L), total bilirubin 6.5 mg/dL (reference 0.1–1.2 mg/dL), direct bilirubin 0.95 mg/dL (reference 0.0–0.3 mg/dL), troponin I 1.240 ng/mL (reference < 0.04 ng/mL), procalcitonin 13.72 ng/mL (reference < 0.05 ng/mL), CRP 48 mg/L (reference < 5 mg/L), INR 2.0 (reference 0.9–1.1), PT 24 s (reference 11–13.5 s), aPTT 58 s (reference 25–35 s), fibrinogen 1.8 g/L (reference 2.0–4.0 g/L), lactate 4.2 mmol/L (reference 0.5–2.2 mmol/L), creatinine 0.96 mg/dL (reference 0.7–1.3 mg/dL), BUN 43 mg/dL (reference 7–20 mg/dL), and pH 7.38 (reference 7.35–7.45). Chest computed tomography pulmonary angiography showed no evidence of pulmonary embolism but revealed bilateral basal consolidative changes with gravity-dependent atelectasis and perifissural fluid, consistent with pulmonary edema and ACS.

Following the second cardiac arrest, hematologic parameters showed severe thrombocytopenia (platelet count 37 × 10^3^/µL; reference 150–400 × 10^3^/µL), white blood cells 7.6 × 10^3^/µL (reference 4.5–11.0 × 10^3^µL), marked LDH elevation (7,169 U/L; reference 140–280 U/L, > 25 × upper limit of normal), total bilirubin 6.8 mg/dL (reference 0.1–1.2 mg/dL), direct bilirubin 1.1 mg/dL (reference 0.0–0.3 mg/dL), troponin I 0.9 ng/mL (reference < 0.04 ng/mL), procalcitonin 26 ng/mL (reference < 0.05 ng/mL), CRP 45.8 mg/L (reference < 5 mg/L), lactic acid 8.23 mmol/L (reference 0.5–2.2 mmol/L), INR 2.19 (reference 0.9–1.1), PT 26 s (reference 11–13.5 s), aPTT 52 s (reference 25–35 s), fibrinogen 2.1 g/L (reference 2.0–4.0 g/L, borderline low), creatinine 1.18 mg/dL (reference 0.7–1.3 mg/dL), BUN 45 mg/dL (reference 7–20 mg/dL), pH 7.17 (reference 7.35–7.45), pO_2_ 55 mm Hg (reference 80–100 mm Hg), pCO_2_ 38 mm Hg (reference 35–45 mm Hg), HCO_3_^–^ 14 mmol/L (reference 22–28 mmol/L), base excess –14 mmol/L (reference –2 to +2 mmol/L), and PaO_2_/FiO_2_ ratio 56 mm Hg (reference > 300 mm Hg). Echocardiography demonstrated left ventricular ejection fraction of 40–45% with global hypokinesis, right ventricular dilation with moderate systolic dysfunction, moderate-to-severe tricuspid regurgitation, and right ventricular systolic pressure of 60 mm Hg, consistent with moderate pulmonary hypertension. Respiratory failure was severe (PaO_2_/FiO_2_ 56 mm Hg on fraction of inspired oxygen 1.0, positive end-expiratory pressure 12 cm H_2_O). Neurologic examination showed Glasgow Coma Scale 12 (E3, V4, M5). Magnetic resonance imaging demonstrated watershed zone ischemic changes in bilateral parietal-occipital regions.

### Diagnosis

The constellation of respiratory failure, thrombocytopenia, bone pain, and marked LDH elevation raised concern for fat embolism syndrome secondary to bone marrow necrosis [13], as well as thrombotic thrombocytopenic purpura like syndrome [14]. Severe ACS with progression to ARDS and septic shock were also considered. The absence of pulmonary embolism on CT angiography and the pattern of bilateral basal consolidation favored ACS with pulmonary edema as the primary driver of respiratory failure, complicated by cardiogenic shock and possible microangiopathic processes. Hemophagocytic lymphohistiocytosis was also considered given the marked LDH elevation and cytopenias. The coagulopathy (INR 2.19, PT 26 s) in the setting of refractory shock and multiorgan hypoperfusion likely reflected a combination of consumptive coagulopathy, hepatic hypoperfusion (shock liver), and systemic inflammatory activation.

The marked thrombocytopenia and LDH elevation in the setting of multiorgan failure created diagnostic uncertainty regarding the relative contributions of vaso-occlusion, hemolysis, fat embolism, and secondary thrombotic microangiopathy. No single test could definitively distinguish among these possibilities. Unfortunately, reticulocyte count and formal peripheral smear examination were not performed during this admission, limiting the ability to differentiate among vaso-occlusion, hemolysis, fat embolism syndrome, and thrombotic microangiopathy. The presence of nucleated red blood cells and a low reticulocyte count would have supported fat embolism syndrome, whereas schistocytes would have indicated thrombotic microangiopathy, requiring empiric management targeting multiple pathophysiologic pathways.

Given two cardiac arrests, refractory shock requiring four vasopressors, severe ARDS, biventricular failure, and severe multiorgan dysfunction, the anticipated mortality was extremely high. Conventional algorithms would strongly support ECMO initiation.

### Treatment

Given refractory hypoxemia, biventricular failure, and two cardiac arrests, a multidisciplinary meeting involving critical care, cardiology, hematology, and cardiac surgery was convened. Initial polling of 10 consultants revealed 80% favored veno-arterial ECMO, 10% favored veno-venous ECMO, and 10% proposed a 12–24 h trial of maximal medical therapy combining RCE, PLEX, and optimized inotropic support prior to ECMO. The risks of ECMO in SCD—including hemolysis, thrombosis, and bleeding—were weighed against the rationale for PLEX in removing inflammatory mediators, cell-free hemoglobin, heme-related mediators, and microthrombi. The final decision was to proceed with aggressive RCE and daily PLEX, with reassessment at 12–24 h. An ECMO circuit was prepared but not initiated.

The patient received conservative crystalloid administration guided by dynamic assessment of fluid responsiveness to avoid right ventricular overload. Volume administration was carefully titrated given the biventricular dysfunction and pulmonary hypertension identified on post-arrest echocardiography.

The therapeutic protocol included RCE exchanging approximately 1–1.5 blood volumes per session every 48 h to target hemoglobin S < 30% and hemoglobin 10 g/dL post-exchange, as per ASFA guidelines [11]. Daily membrane-based PLEX exchanged 1.0–1.5 plasma volumes (3.5–4.5 L) with 5% albumin/saline (80%) and fresh frozen plasma (20%), using citrate-based regional anticoagulation for a total of seven sessions. Concurrent pharmacotherapy included broad-spectrum antibiotics (meropenem, vancomycin), which were initiated after the second cardiac arrest on day 1, hydroxyurea 1,500 mg daily [15], a prednisolone taper, and methotrexate 20 mg weekly per institutional protocol.

Inhaled nitric oxide was discussed by the multidisciplinary team as a potential adjunct for pulmonary vasodilation and right ventricular afterload reduction; however, it was not administered because the combined apheresis strategy was prioritized and the patient demonstrated rapid hemodynamic improvement within hours of initiation.

No major changes to the apheresis protocol were required. Vasopressor support was titrated based on hemodynamic response, with progressive weaning as clinical improvement became apparent. Ventilatory support was adjusted according to lung-protective strategies and patient-ventilator synchrony.

### Follow-up and outcomes

**Figure 1:**
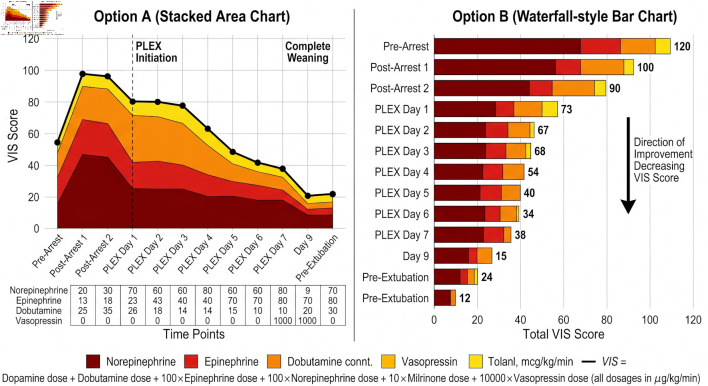
Vasopressor requirements and Vasoactive Inotropic Score (VIS) during recovery from refractory multiorgan failure. PLEX: plasma exchange.

**Figure 2:**
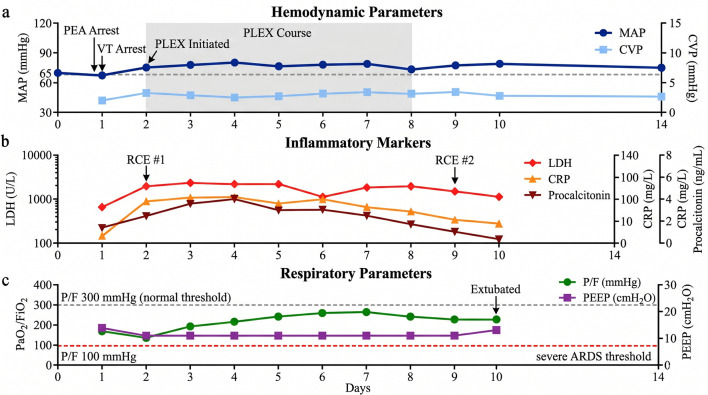
Temporal evolution of hemodynamic (a), inflammatory (b), and respiratory (c) parameters during combined RCE and plasma exchange therapy. ARDS: acute respiratory distress syndrome; CRP: C-reactive protein; CVP: central venous pressure; LDH: lactate dehydrogenase; MAP: mean arterial pressure; P/F ratio: PaO_2_/FiO_2_ ratio; PEA: pulseless electrical activity; PEEP: positive end-expiratory pressure; RCE: red cell exchange; VT: ventricular tachycardia.

**Figure 3:**
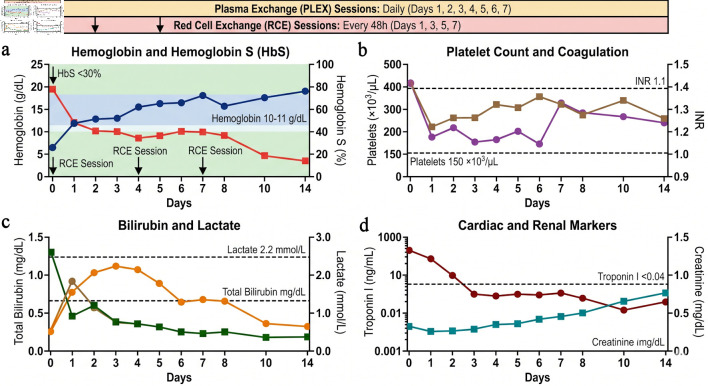
Serial laboratory parameters and clinical response to combined apheresis therapy (a–d). INR: international normalized ratio; RCE: red cell exchange.

Clinical improvement was observed within 48 h. Vasopressor requirements decreased markedly within 24 h of combined apheresis initiation, with complete independence from vasoactive support by day 8 (Tables 2, 3 and Fig. 1). Hemoglobin S was reduced from 73% to 18%. The pH and lactate gradually normalized, and inflammatory markers declined substantially within 72 h (Table 4, Figs. 2, 3).

**Table 2:** Vital Signs and Hemodynamic Parameters

Time point	Temperature (°C)	HR (/min)	BP (mm Hg)	MAP (mm Hg)	CVP (mm Hg)
Admission	37.2	95	128/78	95	8
Pre-arrest	38.5	118	85/52	68	14
Post-arrest 1	36.8	145 (AF)	68/42	52	18
Post-arrest 2	35.5	165 (VT)	62/38	48	22
PLEX day 1	37.0	95 (SR)	75/48	58	16
PLEX day 2	37.2	88 (SR)	82/55	65	14
PLEX day 3	37.1	82 (SR)	95/62	72	12
PLEX day 5	36.9	78 (SR)	105/68	78	10
PLEX day 7	36.8	76 (SR)	115/72	85	8
Pre-extubation	36.8	72 (SR)	118/75	88	8
ICU discharge	36.7	68 (SR)	125/78	92	6

HR: heart rate; BP: blood pressure; MAP: mean arterial pressure; CVP: central venous pressure; AF: atrial fibrillation; VT: ventricular tachycardia; SR: sinus rhythm; PLEX: plasma exchange; ICU: intensive care unit.

**Table 3:** Detailed Vasopressor Administration and Titration

Time point	Norepinephrine (µg/kg/min)	Epinephrine (µg/kg/min)	Vasopressin (units/min)	Dobutamine (µg/kg/min)	Total VIS
Pre-arrest (day 1, 8:00 am)	0.15	0	0	0	15
Post-arrest 1 (day 1, 2:00 pm)	0.45	0.15	0.04	5.0	75
Post-arrest 2 (day 2, 2:00 am)	0.65	0.25	0.04	8.0	108
PLEX day 1 (day 2, 8:00 am)	0.35	0.12	0.04	5.0	62
PLEX day 2 (day 3, 8:00 pm)	0.28	0.08	0.04	3.0	49
PLEX day 3 (day 4, 8:00 am)	0.18	0.06	0.03	2.0	33.5
PLEX day 4 (day 5, 8:00 pm)	0.12	0.03	0.02	1.0	21
PLEX day 5 (day 6, 8:00 am)	0.08	0	0.02	0	13
PLEX day 6 (day 7, 8:00 pm)	0.05	0	0	0	5
PLEX day 7 (day 8, 8:00 am)	0.04	0	0	0	4
Day 9 (8:00 am)	0	0	0	0	0
Pre-extubation (day 10)	0	0	0	0	0
ICU discharge (day 14)	0	0	0	0	0

Vasoactive Inotropic Score (VIS) was calculated as: norepinephrine (µg/kg/min × 100) + epinephrine (µg/kg/min × 100) + dobutamine (µg/kg/min × 1) + vasopressin (units/min × 10,000). PLEX: plasma exchange; ICU: intensive care unit.

**Table 4:** Selected Laboratory and Physiologic Parameters

Parameter	Admission/day 0	Pre-arrest/day 1	Post-PLEX/day 1	Post-PLEX/day 3	Pre-extubation/day 10	ICU discharge/day 14
Hemoglobin (g/dL)	7.8	7.7	11.4	9.6	9.9	10.0
Hemoglobin S (%)	73	73	35	18.7	20	18
Platelets (× 10^3^/µL)	145	84	37	51	76	145
WBC (× 10^3^/µL)	12.3	12.7	7.6	8.1	6.4	6.6
LDH (U/L)	2,150	3,855	7,169	4,583	1,747	827
Total bilirubin (mg/dL)	4.2	6.5	6.8	2.8	2.3	1.3
Direct bilirubin (mg/dL)	0.9	0.95	1.1	0.64	0.53	0.47
Troponin I (ng/mL)	0.04	1.240	0.9	0.45	0.12	0.05
Procalcitonin (ng/mL)	2.1	13.72	26	16.4	5.8	0.8
CRP (mg/L)	16	48	45.8	12.44	4.59	2.4
INR	1.2	2.0	2.19	1.76	1.31	1.4
PT (s)	12	24	26	15.8	13.5	12.8
aPTT (s)	32	58	52	45	35	32
Fibrinogen (g/L)	3.2	1.8	2.1	2.4	3.8	4.1
Creatinine (mg/dL)	0.67	0.96	1.18	0.82	0.62	0.54
BUN (mg/dL)	28	43	45	37	21	21
Lactate (mmol/L)	1.2	8.23	5.2	2.4	1.6	1.6
pH	7.38	7.17	7.45	7.52	7.52	7.44
pO_2_ (mm Hg)	78	55	66	88	209	88
pCO_2_ (mm Hg)	42	38	52	42	41	40
HCO_3_^–^ (mmol/L)	24	14	26	33	30	28
Base excess (mmol/L)	+1	−14	+1	+5	+4	+2
PaO_2_/FiO_2_ ratio	280	56	86	195	491	303

Reference ranges: hemoglobin 13.5–17.5 g/dL (male); platelets 150–400 × 10^3^/µL; WBC 4.5–11.0 × 10^3^/µL; LDH 140–280 U/L; total bilirubin 0.1–1.2 mg/dL; direct bilirubin 0.0–0.3 mg/dL; troponin I < 0.04 ng/mL; procalcitonin < 0.05 ng/mL; CRP < 5 mg/L; INR 0.9–1.1; PT 11–13.5 s; aPTT 25–35 s; fibrinogen 2.0–4.0 g/L; creatinine 0.7–1.3 mg/dL; BUN 7–20 mg/dL; lactate 0.5–2.2 mmol/L; pH 7.35–7.45; pO_2_ 80–100 mm Hg; pCO_2_ 35–45 mm Hg; HCO_3_^–^ 22–28 mmol/L; base excess −2 to +2 mmol/L; PaO_2_/FiO_2_ > 300 mm Hg. aPTT: activated partial thromboplastin time; BUN: blood urea nitrogen; CRP: C-reactive protein; FiO2: fraction of inspired oxygen; HCO3–: bicarbonate; INR: international normalized ratio; LDH: lactate dehydrogenase; PaO2: partial pressure of oxygen in arterial blood; PT: prothrombin time; WBC: white blood cell.

Despite initial Glasgow Coma Scale 12 and magnetic resonance imaging demonstrating watershed zone ischemic changes in bilateral parietal-occipital regions, the patient achieved full neurologic recovery by intensive care unit discharge (GCS 15/15, intact executive function, no motor deficits). Repeat echocardiography on day 10 showed left ventricular ejection fraction of 55% (normalized), improved right ventricular function with mild dilation only, and right ventricular systolic pressure of 31 mm Hg (normalized from 60 mm Hg). Respiratory support progressed from pressure-controlled continuous mandatory ventilation (PaO_2_/FiO_2_ 33) to successful extubation on day 10 (PaO_2_/FiO_2_ 491 on pressure support ventilation), with transition to room air by discharge (PaO_2_/FiO_2_ 419) (Table 5, Fig. 2). Platelet count normalized by day 10 (Table 4, Fig. 3). The patient was discharged from the intensive care unit on day 14 after a 16-day hospitalization.

**Table 5:** Respiratory and Ventilatory Parameters

Phase	Mode	FiO_2_	PEEP (cm H_2_O)	pH	PaO_2_ (mm Hg)	PaCO_2_ (mm Hg)	P/F ratio
Pre-ICU (BiPAP)	BiPAP	0.60	12/8	7.35	65	48	108
Post-arrest	PC-CMV	1.00	12	7.16	33	57	33
PLEX day 1	PC-CMV	0.80	10	7.28	52	48	65
PLEX day 2	PRVC	0.60	10	7.35	72	45	120
PLEX day 3	PCV	0.45	8	7.42	88	42	195
PLEX day 5	PCV	0.40	6	7.47	95	38	237
Pre-extubation	PSV	0.35	5	7.52	172	42	491
Post-extubation	NC	0.28	—	7.48	85	40	303
Hospital discharge	RA	0.21	—	7.44	88	40	419

BiPAP: bilevel positive airway pressure; PC-CMV: pressure-controlled continuous mandatory ventilation; PCV: pressure control ventilation; PRVC: pressure regulated volume control; PSV: pressure support ventilation; NC: nasal cannula; RA: room air; PEEP: positive end-expiratory pressure; FiO_2_: fraction of inspired oxygen; ICU: intensive care unit; P/F ratio: PaO_2_/FiO_2_ ratio; PaCO_2_: partial pressure of carbon dioxide in arterial blood; PaO_2_: partial pressure of oxygen in arterial blood; PLEX: plasma exchange.

Formal patient-reported outcome measures were not collected during the acute illness due to the severity of presentation and need for mechanical ventilation. However, by intensive care unit (ICU) discharge, the patient reported resolution of bone pain and dyspnea and was able to ambulate with assistance.

The patient tolerated all seven PLEX sessions and intermittent RCE without procedure-related complications, bleeding, or need for transfusion beyond the exchange protocol. No adverse events related to apheresis were observed. No bleeding complications, catheter-related infections, or citrate toxicity occurred.

## Discussion

This case illustrates the potential role of combined RCE and PLEX in fulminant SCD crisis with refractory multiorgan failure. The patient presented with two cardiac arrests, severe ARDS, biventricular dysfunction, and marked inflammatory markers clinical features that would typically prompt ECMO initiation. Instead, a strategy of intensive apheresis was associated with rapid hemodynamic stabilization, resolution of vasopressor dependence, and ultimately full recovery.

This case exemplifies the paradigm that severe SCD complications represent a systemic inflammatory and thrombotic microangiopathy rather than purely mechanical vaso-occlusion [16, 17]. The progression from vaso-occlusive crisis to ACS, shock, cardiac arrest, and multi-organ dysfunction reflects escalating endothelial activation, complement consumption, coagulation cascade activation, and cytokine storm [16, 17]. Hemolysis-driven vasculopathy occurs when the release of cell-free hemoglobin scavenges nitric oxide, thereby impairing vascular relaxation and promoting the expression of endothelial adhesion molecules [18]. Simultaneously, platelet activation and microthrombosis are fueled by ischemia-reperfusion injury, which triggers both platelet aggregation and the formation of neutrophil extracellular traps (NETs) [19, 20]. These processes feed into a systemic inflammatory cascade, where interleukin (IL)-1β, IL-6, IL-8, and tumor necrosis factor-alpha (TNF-α) drive a systemic inflammatory response syndrome (SIRS)-like pathology [20, 21]. Finally, fat embolism syndrome—stemming from bone marrow necrosis releases fat globules and pro-inflammatory mediators into the circulation, manifesting in a clinical picture similar to thrombotic thrombocytopenic purpura [13].

Some observations from this case warrant consideration. First, while RCE remains the cornerstone of severe ACS management [4], optimal hematological correction (hemoglobin S 18%) did not immediately precede clinical improvement; rather, the most rapid hemodynamic gains coincided with the initiation of daily PLEX. The pathophysiology of fulminant SCD involves endothelial activation, inflammation, and coagulopathy [11, 16, 17], and PLEX may directly remove circulating cytokines [11], cell-free hemoglobin and heme-related mediators [22], and prothrombotic factors including ultra-large von Willebrand factor multimers [11]. This temporal association raises the hypothesis that such mechanistic clearance may complement rheological correction in severe SCD [11, 12].

Second, the marked LDH elevation and severe thrombocytopenia suggested a process beyond simple vaso-occlusion, such as fat embolism syndrome or secondary thrombotic microangiopathy [13, 14], conditions in which RCE alone may be insufficient [13, 23]. The precipitous decline in LDH (7,169 U/L peak to 827 U/L at discharge, 88% reduction) and procalcitonin (26 to 0.8 ng/mL) suggests rapid attenuation of cell destruction and inflammatory signaling. While procalcitonin rose markedly (2.1 to 26 ng/mL), we emphasize that it was interpreted as a biomarker of systemic inflammatory severity and hemolysis rather than as a decision tool for antibiotic initiation; empiric antibiotics were commenced after the second cardiac arrest based on clinical suspicion of sepsis in the setting of fulminant multiorgan failure, not on procalcitonin values alone.

Third, the rapid deterioration on day 1 raises the question of whether earlier initiation of exchange transfusion and antibiotics could have altered the clinical course. The patient received standard ward-level supportive care on admission, with escalation to intensive care only after overt respiratory decompensation. In retrospect, given the patient’s history of recurrent ACS and rapidly progressive symptoms, earlier transfer to intensive care and pre-emptive RCE might be considered. However, the fulminant progression from stable ward status to cardiac arrest within hours suggests an unusually severe phenotype that may not have been preventable with standard timelines of care. The initiation of antibiotics after the second arrest, rather than on admission, represents a potential delay that may have contributed to initial worsening; we have revised the text to clarify this timeline and acknowledge this limitation.

Fourth, regarding the sequence of interventions, combined RCE and daily PLEX were initiated simultaneously after the second cardiac arrest as part of a pre-defined multidisciplinary salvage protocol, rather than as sequential rescue after failure of RCE alone. This simultaneous approach was chosen given the severity of multiorgan failure and the mechanistic rationale for combined cytokine and rheological correction. RCE would decrease sickling, reduce pulmonary vascular resistance, and offload the right ventricle with improvement in hemodynamics regardless of PLEX; however, the rapid clinical response suggests synergism between the two modalities.

PLEX offers mechanistic complementarity to RCE through cytokine removal, utilizing high-cutoff membranes or centrifugation to eliminate IL-6, IL-8, TNF-α, and other inflammatory mediators within the 15–25 kDa range [24, 25]. It further targets pathological proteins, removing ultra-large von Willebrand factor multimers, free hemoglobin, and haptoglobin-hemoglobin complexes [14]. Additionally, PLEX promotes endothelial stabilization by clearing circulating microparticles and adhesion molecules [26], facilitates fat emboli clearance (specifically lipid particles and chylomicron remnants) [13], and provides complement modulation by reducing activated components such as C3a and C5a [27]. The daily PLEX regimen for 7 consecutive days provided sustained clearance of pathological mediators during the critical recovery period.

The role of ECMO in SCD deserves contextualization. Registry analyses of 206 adult SCD patients receiving ECMO report overall in-hospital survival of 40.3%, with venoarterial ECMO survival at 25.5% versus venovenous ECMO at 61.1% [9]. Hemorrhage occurred in 26% of patients, neurological injury in 22%, and thrombosis in 13% [9, 10]. Bleeding and thrombotic events are particularly common, occurring in up to 45.6% of venoarterial ECMO and 33.8% of venovenous ECMO runs [9]. These complications are particularly concerning in SCD, where hemolysis, thrombophilia, and endothelial dysfunction are already present [16, 17]. This evidence supports ECMO as rescue therapy rather than first-line intervention [8], with the caveat that the window for disease-specific therapy may narrow if mechanical support is delayed excessively. The 12–24 h “medical optimization” window proposed here requires intensive monitoring, prompt disease-specific interventions, and immediate ECMO availability for refractory cases.

This case contributes to emerging literature on PLEX as adjunctive therapy in severe SCD. A recent systematic review identified PLEX as a potentially beneficial intervention in SCD with multiorgan failure, though data remain limited to case series and small cohorts [11]. Accumulating reports, including case series describing reversal of severe multiorgan failure with PLEX in SCD crises resistant to RCE alone [11, 22, 23], support a potential role for intensive apheresis in selected patients.

Based on this case and literature synthesis, we propose a structured disease-specific escalation algorithm for refractory SCD crises. Key principles include: (1) Early apheresis: Do not delay PLEX until “failure” of RCE alone; (2) Biomarker-guided therapy: LDH and inflammatory markers can be used to monitor treatment response; (3) ECMO as bridge, not treatment: Must continue disease-specific therapy during ECMO if utilized; and (4) Hematology–critical care integration: Mandatory multidisciplinary decision-making is essential.

Strengths include the detailed physiologic and laboratory documentation, the clear temporal association between intervention initiation and clinical response, the multidisciplinary decision-making process, and the comprehensive hemodynamic and respiratory parameter tracking. Limitations must be acknowledged. First, this is a single case report; no causal inference can be drawn regarding the specific contribution of PLEX versus RCE, supportive care, or the natural disease course. Second, multiple interventions were administered simultaneously, precluding isolation of any single treatment effect. Third, survivor bias may influence the apparent efficacy of the approach. Fourth, the generalizability of this strategy to other patients with SCD and refractory failure is unknown. Fifth, the absence of reticulocyte count and peripheral smear limits definitive differential diagnosis among hemolysis, fat embolism syndrome, and thrombotic microangiopathy. Sixth, the delay in antibiotic initiation and exchange transfusion may have contributed to the fulminant course, and earlier escalation should be considered in future cases. Finally, formal patient-reported outcomes were not collected during the acute phase.

Learning points include: (1) Combined RCE and daily PLEX may represent a viable salvage strategy for post-arrest refractory cardiorespiratory failure in SCD, potentially sparing the need for ECMO in carefully selected patients; (2) The rapid hemodynamic and neurologic recovery observed here supports further prospective investigation of intensive apheresis protocols for severe SCD complications; (3) Future research should prioritize prospective registries, randomized trials comparing RCE + PLEX to RCE alone in severe ACS, and mechanistic studies of plasma proteins to identify novel therapeutic targets.

## Data Availability

All data generated or analyzed during this study are included in this published article. Additional deidentified clinical data are available from the corresponding author upon reasonable request.

## Abbreviations

ACS: acute chest syndrome
AF: atrial fibrillation
aPTT: activated partial thromboplastin time
ARDS: acute respiratory distress syndrome
ASFA: American Society for Apheresis
BiPAP: bilevel positive airway pressure
BUN: blood urea nitrogen
CMV: continuous mandatory ventilation
CPR: cardiopulmonary resuscitation
CRP: C-reactive protein
CTPA: computed tomography pulmonary angiography
CVP: central venous pressure
ECMO: extracorporeal membrane oxygenation
ELSO: extracorporeal life support organization
FES: fat embolism syndrome
FFP: fresh frozen plasma
FiO_2_: fraction of inspired oxygen
G6PD: glucose-6-phosphate dehydrogenase
GCS: Glasgow Coma Scale
HbS: hemoglobin S (sickle hemoglobin)
HbSS: homozygous sickle cell genotype
HCO_3_^–^: bicarbonate
HLH: hemophagocytic lymphohistiocytosis
ICU: intensive care unit
IL: interleukin
INR: international normalized ratio
LDH: lactate dehydrogenase
LVEF: left ventricular ejection fraction
MAP: mean arterial pressure
MOF: multi-organ failure
mri: magnetic resonance imaging
NC: nasal cannula
NETs: neutrophil extracellular traps
NO: nitric oxide
P/F ratio: PaO_2_/FiO_2_ ratio
PaCO_2_: partial pressure of carbon dioxide in arterial blood
PaO_2_: partial pressure of oxygen in arterial blood
PC-CMV: pressure-controlled continuous mandatory ventilation
PCT: procalcitonin
PCV: pressure control ventilation
PEA: pulseless electrical activity
PEEP: positive end-expiratory pressure
PLEX: plasma exchange
PRVC: pressure regulated volume control
PSV: pressure support ventilation
PT: prothrombin time
RBC: red blood cell
RCE: red cell exchange
ROSC: return of spontaneous circulation
RV: right ventricular
RVSP: right ventricular systolic pressure
SCD: sickle cell disease
SIRS: systemic inflammatory response syndrome
SpO_2_: peripheral oxygen saturation
SR: sinus rhythm
SVT: supraventricular tachycardia
TNF-α: tumor necrosis factor-alpha
TTP: thrombotic thrombocytopenic purpura
VA-ECMO: veno-arterial extracorporeal membrane oxygenation
VIS: Vasoactive Inotropic Score
VOC: vaso-occlusive crisis
VV-ECMO: veno-venous extracorporeal membrane oxygenation
vWF: von Willebrand factor
WBC: white blood cell
